# Development of hybrid coarse-grained atomistic models for rapid assessment of local structuring of polymeric semiconductors

**DOI:** 10.1039/d1me00165e

**Published:** 2022-01-07

**Authors:** Maryam Reisjalali, Rex Manurung, Paola Carbone, Alessandro Troisi

**Affiliations:** Department of Chemistry, University of Liverpool Crown St L69 7ZD Liverpool UK m.reisjalali@liverpool.ac.uk a.troisi@liverpool.ac.uk; Department of Chemical Engineering and Analytical Science Oxford Road M13 9PL Manchester UK paola.carbone@manchester.ac.uk

## Abstract

Decades of work in the field of computational study of semiconducting polymers using atomistic models illustrate the challenges of generating equilibrated models for this class of materials. While adopting a coarse-grained model can be helpful, the process of developing a suitable model is particularly non-trivial and time-consuming for semiconducting polymers due to a large number of different interactions with some having an anisotropic nature. This work introduces a procedure for the rapid generation of a hybrid model for semiconducting polymers where atoms of secondary importance (those in the alkyl side chains) are transformed into coarse-grained beads to reduce the computational cost of generating an equilibrated structure. The parameters are determined from easy-to-equilibrate simulations of very short oligomers and the model is constructed to enable a very simple back-mapping procedure to reconstruct geometries with atomistic resolution. The model is illustrated for three related polymers containing DPP (diketopyrrolopyrrole) to evaluate the transferability of the potential across different families of polymers. The accuracy of the model, determined by comparison with the results of fully equilibrated simulations of the same material before and after back-mapping, is fully satisfactory for two out of the three cases considered. We noticed that accuracy can be determined very early in the workflow so that it is easy to assess when the deployment of this method is advantageous. The hybrid representation can be used to evaluate directly the electronic properties of structures sampled by the simulations.

## Abstract 2

A model of semiconducting polymers where the conjugated portion is described with atomistic detail and the alkyl side chains are approximated with a coarse grain potential is very promising for the molecular design of new polymeric materials.

## Graphical abstract

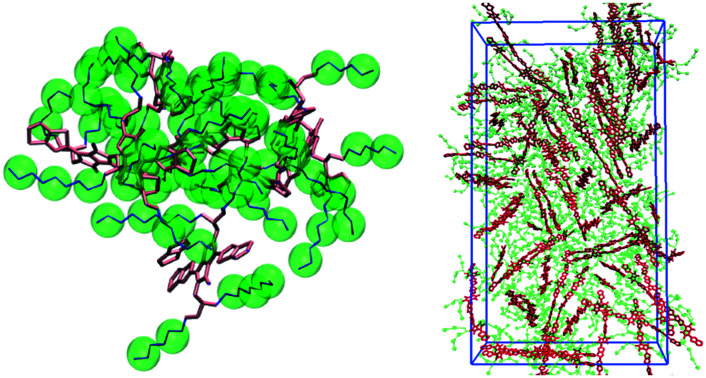

**Design, System, Application**

The goal of organic electronics is to explore the rich set of tools of synthetic chemistry to generate materials with specific electronic functions. This paradigm has not been fully realized in the area of polymeric semiconductors because, it is now clear, their electronic properties are determined by their local structuring, a feature that is very difficult to predict and design. Molecular simulations give substantial insight and can offer the key to facilitate design and optimization of semiconducting polymers. At the moment, however, they are too slow to be deployed in the design phase and they are mostly limited to the characterization of well-studied benchmark materials. To unlock the potential of molecular simulations in the design of new materials it is essential to have a rapid and accurate method that can be deployed in principle to a large number of structures. We explore in this contribution the adoption of a hybrid atomistic and coarse grained model that produces an accurate representation of the local ordering of the polymer while retaining the information required to evaluate its electronic properties.

## 1 Introduction

Semiconducting polymers (SCP) are one of the most studied classes of soft functional materials with a range of applications that have evolved substantially from the first applications in transistors^1,2^ and solar cells^3–6^ to new emerging areas like neuromorphic computing,^7–9^ bioelectronics^10–12^ and photocatalysis.^13,14^ Since the early finding of the local structuring of an SCP determining its electronic properties and ultimately its functionality,^15–18^ the modelling community has contributed to the formulation of microscopic models of the polymer that could explain the observations and direct the development of new materials. Very detailed features of the polymers, like the mutual orientation of the conjugated fragments, are essential in determining its structure and the initial goal has been to perform all-atomistic (AA) classical simulations where the position of all atoms is described.^19–21^

The number of semiconducting polymers for which AA simulations have been conducted is a very small fraction of those that have shown very promising characteristics simply because of the labour and computational cost required. While appropriate for benchmark polymers, the AA approach is hardly scalable for the study of a large number of polymers, for example, if one is interested in designing materials by studying hypothetical polymers. Many authors have discussed the challenge of generating an equilibrated structure for SCPs. For example, Carrillo *et al.*^22^ show that performing simulated annealing for a shorter time frame does not produce an equilibrated morphology by studying different morphologies achieved using different timescales and system sizes. Moreno *et al.*^23^ in their study report that, despite maximum efforts in making sure the most appropriate force-field (FF) was chosen for a set of polythiophenes, there remains a degree of side chain disorder at room temperature. Many attempts are made to optimise simulation performances for different models of AA simulations, such as using implicit solvent model,^24^ the approximation of rigid monomer units^25,26^ and the study of polymers at higher temperatures.^27^ However, these approximations can be unsatisfactory where the solvent has an active role in the assembly/chain conformations, or where the polymeric system's morphology is dependent on chain flexibility.^28^

One of the most common strategies used in polymer simulation to accelerate the construction of an equilibrated model is based on the idea of coarse-graining (CG) where many atom groups are clustered into a virtual bead.^29–31^ Using CG methods has made it possible to study processes at scales comparable to the active layer of photovoltaics, like the migration of shorter polymer chains toward the donor–acceptor interface reported by Carrillo *et al.*^32^ These studies, often validated against selected experimental data,^33^ also enable the use of morphologies of the CG simulations to back-map to an AA representation for further studies at higher resolutions.^34–38^ A benchmark example, revisited by different authors, is the development of CG force fields for common polythiophenes such as P3HT,^34,39,40^ and, more recently, the goal has shifted toward the direct prediction of electronic properties from mesoscale simulation.^41,42^ A particular challenge for the development of a CG potential for this class of materials is that the most common procedures for generating such potentials such as iterative Boltzmann inversion or inverse Monte Carlo methods^43–45^ require iterative processes to define a numerical potential between any pair of beads. The complexity of the iterative process and the risk of non-convergence increase as the number of bead types increases and becomes inconvenient for the complex polymers of contemporary interest. Another reason for a lower amount of research on SCPs using CG compared to AA is the anisotropic nature of the relevant interactions. It is common in most CG models to assume isotropic inter-bead interactions while the intermolecular interactions in a SCP dictate the structure and morphological characteristics of these materials. Essentially all polymers consist of a conjugated backbone and more flexible side chains with a strong tendency for the conjugated backbone to form π–π stacking. Therefore, models are needed where anisotropic interactions are included to correctly reproduce the π–π interaction.^46–49^

A possible alternative to either of AA or CG approach, which is explored in this article, is to develop a suitable hybrid model in which atoms of secondary importance to the function of the materials (in this case the alkyl side chains) are coarse-grained while the rest of the system (in this case the conjugated backbone) is kept with an atomic resolution. This AA–CG approach will naturally avoid the issue of anisotropic interactions between π-conjugated fragments (because they are kept at an atomistic resolution) and might also be able to preserve the correct polymer dynamics^50^ overcoming the well-known problem of fast dynamics of CG models. The side chains of polymeric systems may be described with a single type of CG bead making the parametrization of the potential substantially quicker with a cost that does not increase when the complexity of the polymer backbone increases. Furthermore, the similarity of side chains across different polymers may allow the developed CG FF at least partially transferable to other polymers. Another potential advantage of being able to have a CG representation of the side chains is the possibility of performing the integration of the equation of motion with a larger integration time step if the individual monomers are treated as rigid units.^24^ Hybrid models have been proposed in different contexts^29,51,52^ with a prevalence of applications in biological simulations such as nucleic acids^35,53^ and membrane^54^ as well as polymers including polyethylene glycol, polystyrene and octanol.^55–58^ However, the choice and definition of acceptable approximations differ for each of the mentioned examples due to their different motivations of study. For example, Gower *et al.*^59^ developed a hybrid model specifically to investigate the role of H-bonding in a large model of polyamide where the long-range mechanical properties are influenced by short-range interaction. Hybrid models, however, have not been used so far to accelerate the exploration of larger sets of materials.

The goal of this paper is to establish the accuracy of a rapid and general method based on hybrid AA–CG simulations to generate equilibrated models of semiconducting polymers. We consider the accuracy of the suggested approach by comparing against a full AA simulation either (I) the AA–CG model or (ii) a model obtained by back-mapping the atomistic description of atoms into the hybrid model. We apply the procedure to a selection of oligomers containing the diketopyrrolopyrrole (DPP) unit and similar side chains, which have been studied with an AA model in a previous work^27^ and are interesting for their high charge mobility.^5,60^ We specifically explore the transferability of this potential which would enable the rapid application to polymers with different backbones. This approach, while not constrained to any pre-defined beads like the standard MARTINI model,^61–64^ is based on simpler analytical potentials that do not require long rounds of iterations to optimise the parameters, like the Iterative Boltzmann Inversion method.^43^ The proposed procedure reduces the human and computational cost for performing this type of multiscale simulations with an expected reduction in accuracy that this work aims to quantify.

## 2 Methods

### 2.1 Atomistic models

**Fig. 1:**
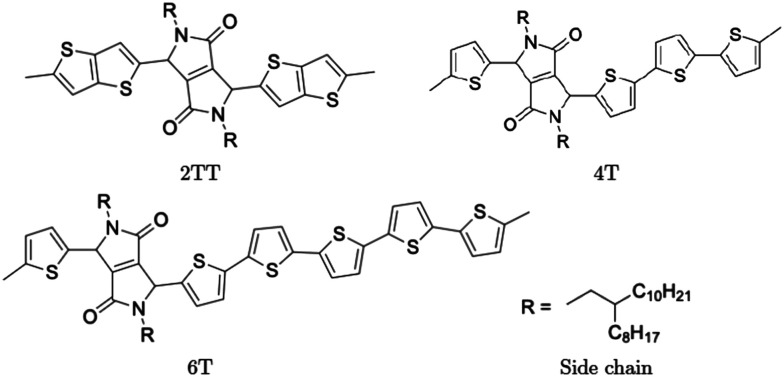
Chemical structure of monomer units and their common side chain.

Polymers belonging to the DPP family of materials were chosen as a set of benchmarking systems to study morphological characteristics and aggregation properties in semiconducting polymers. This work focuses on the development of a hybrid AA–CG model by parameterising an adaptable FF based on systems containing monomers presented in Fig. 1 and labelled as 2TT, 4T and 6T. Atomistic simulations of 64 trimers of each system have been reported in a previous study with details given there on the development of the force field and the description of the local structure obtained after equilibration including links with available.^27^ These simulations are used as a reference to check the accuracy of the hybrid model. The hybrid model, however, is developed from simulations of much smaller systems containing monomers only (also 64 in number), which equilibrates very rapidly. In this way, we can establish early on the accuracy of the workflow, as the simulations needed to generate the potential are computationally inexpensive. It may be worth noticing that the monomers are relatively large in comparison to more common polymers (the smallest has 140 atoms) and that the side chains to be coarse-grained are connected to the central portion of such monomers (Fig. 1).

The FF used for the atomistic simulations is based on the mathematical expressions of OPLS-AA.^65^ As discussed in ref. 27, the parameters for equilibrium bond distance and angle, torsional potential between conjugated fragments and the point charges have been computed from DFT calculations. The experimental information on the local structure is insufficient for a direct comparison with the simulation but the correct trends are reproduced by the atomistic simulation of the π-stacking distance^66^ and the changes in glass transition temperature with increase the number of thiophene rings.^67^ Simulations were performed using the LAMMPS^68^ (large-scale atomic/molecular massively parallel simulator) software. Periodic boundary conditions (PBC) were applied in all directions and the velocity Verlet algorithm was adopted for all simulations. Nosé–Hoover isothermal–isobaric barostat and thermostat were used to control the ensemble properties. All X–H bonds were constrained in the systems of oligomers using a shake/rattle algorithm to enable the use of a larger integration time step of 2 fs and the Ewald electrostatic summation algorithm with the cutoff of 12 Å.

### 2.2 Hybrid AA–CG model

**Fig. 2:**
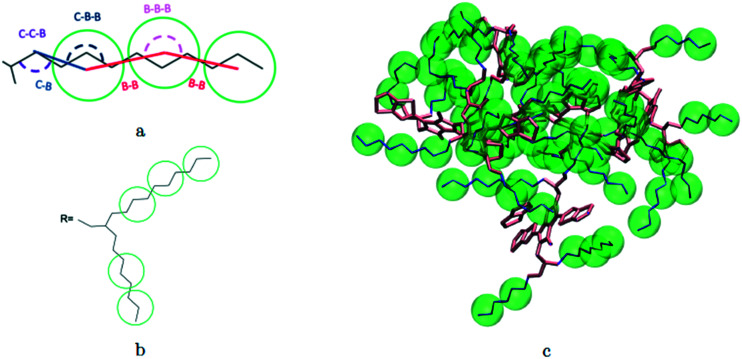
Bead mapping schematic for 2TT as an example system with atomic representations in lines and beads in green spheres. a) Extra bonded force-field parameters introduced for the hybrid model; b) bead mapping along a side chain; c) a sample snapshot. Hydrogens are omitted for simplicity.

A hybrid model was constructed on systems containing 64 monomers represented in Fig. 1 by keeping all atomistic details of the backbone of the molecules and clustering groups of atoms containing 3 carbon atoms in the side chain into individual beads, as illustrated in Fig. 2a and b. The beads are positioned in the geometric centre of three CH_2_ groups. By the addition of beads, labelled B, into the systems, some new interactions must be accounted for in the new hybrid FF which are evaluated differently for the bonded and the non-bonded part (all other details are identical to those used for the atomistic simulations).

The additional bonded parameters required are represented in Fig. 2a and include the bond stretching parameters for the interactions C–B (between a carbon atom and a bead) and B–B (between two beads) as well as three additional bonds bending interaction (C–C–B, C–B–B and B–B–B) represented in Fig. 2a. The bonded (stretch or bend) parameters were derived from the AA simulations of monomers from which snapshots of the simulation are extracted and mapped into hybrid topologies with side chain atoms replaced by beads. Using the new topologies, distributions were calculated for each of the new bond and angle types.

For the intramolecular potentials, we used the common approach of approximating the interaction to a harmonic potential as:^48,69–71^

1 *U*_(bond,*IJ*)_(*r*) = *K*_*IJ*_(*r* − *r*_0,*IJ*_)^2^

2 *U*_(ang,*IJL*)_(*θ*) = *K*_*IJL*_(cos(*θ*) − cos(*θ*_0,*IJL*_))^2^

where the indexes *I*, *J* (and *L*) indicate atom types, *K*_*IJ*_ and *K*_*IJL*_ the bond and angle force constant respectively, *r*_0,*IJ*_ and, *θ*_0,*IJL*_ the equilibrium bond and angle respectively. In the harmonic approximation, the equilibrium angle and distances were the averages of the distributions, and the force constants relate to the standard deviation of the distribution as:3
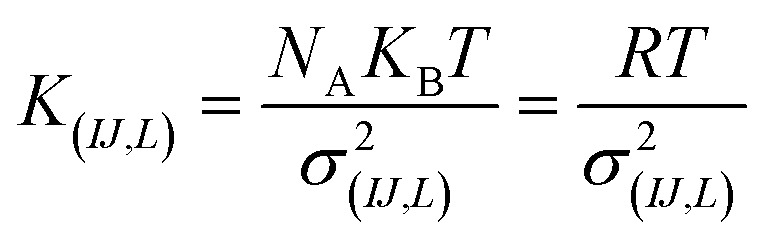
where *N*_A_ is the Avogadro constant, *K*_B_ is Boltzmann's constant, *R* is gas constant, *K*_(*IJ*,*L*)_ is force constant and, *σ*_(*IJ*,*L*)_ is the standard deviation of the bonding distance or angle distribution. The torsional potentials in the CG part were set to zero as the weak torsional potential between beads generates a very flat distribution of dihedral angles often seen for similar systems.^64^

For the non-bonded interaction between beads, we adopted the analytical 6–12 Lennard–Jones (LJ) pair potential4
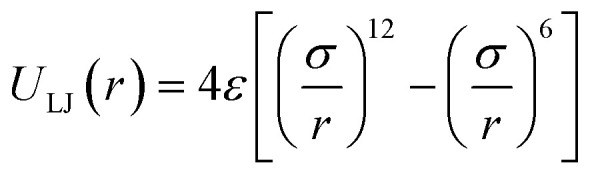
used for example also for the MARTINI model^40^ including recent SCP simulations^72^ Using the Lorentz–Berthelot combination rule the same parameters and functional form is used to describe the interaction between beads and the particles with atomistic representation.

In essence, there are just two non-bonded parameters (*σ* and *ε*) for all non-bonded interaction making the hybrid model easy to parametrize, possibly at the expense of reduced accuracy. We started by considering a range of reasonable *ε* values between 0.6 and 0.95 kcal mol^−1^ by comparison with similar beads in the MARTINI model. For each *ε* a set of 20 ns simulations were performed for different values of *σ* in a fixed increment of 0.05 Å to find (*σ*, *ε*) pairs for which the systems had a density closest to that of the AA (interpolation was used to find the (*σ*, *ε*) pair where the density was identical to the target). This process yielded a set of parameters that would produce a hybrid model with the same density as the AA simulation. Amongst those specific parameters, one can be chosen that best represents the AA model with a second target property. Since the goal is to develop a hybrid method that reliably reproduces the atomistic arrangement of the conjugated backbone, the parameter set was selected as the one that best reproduces the radial distribution function (RDF) of the backbone atom types, as discussed in the results session.

### 2.3 Back-mapping

**Fig. 3:**
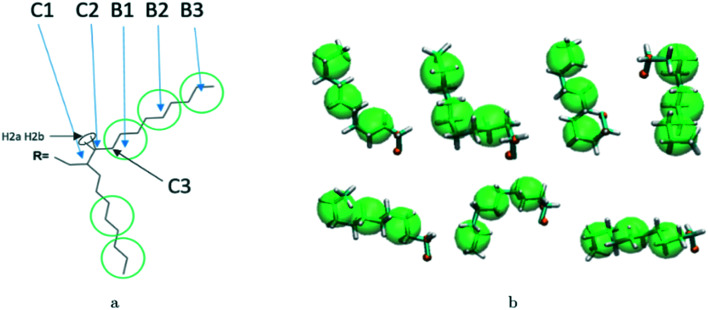
a) Schematic of back-mapping; b) several example chains being back-mapped with atomic details and imposed beads in green spheres.

Reconstructing a model with all atomic coordinates from a hybrid simulation can be useful to validate the hybrid model (*i.e.* no major structural change should take place) and to improve the accuracy of the model (*i.e.* a short equilibrated structure obtained from a back-mapping procedure should be a realistic representation of the equilibrated AA structure). The method to find the coordinates of all C and H atoms previously described by a coarse-grained bead is illustrated with the help of Fig. 3a. The input consists of the cartesian coordinates of the beads B1–B3 (with the same parameters) and coordinate of the atoms C1 and C2 (need to ensure a good connection with atoms C3). After finding the coordinates of C3, the new position for the hydrogen atoms H2a and H2b are computed to make them consistent in relation to the reconstructed atomistic part.

A complete set of 6563 reference conformations for the fragment C–(CH_2_)_9_–CH_3_ was generated by varying all CCCC dihedral angles in discrete steps of 120 degrees starting from 60 degrees (*i.e.* the 3 stable configurations of each dihedral) and excluding the conformations with too short atomic distances. Other bond angles, distances, dihedrals have been set to the equilibrium value of the force field. For each conformation, the corresponding CG conformation (*i.e.* the coordinates of 2 atoms and 3 beads) was constructed producing a library of 6563 paired atomistic and CG representations of possible conformations of the fragment. Given the input coordinates of C1, C2, B1–B3, from the hybrid simulation, they are rotated and translated to minimize the root mean square deviation (RMSD) from each of the reference CG conformations using the Kabsch algorithm.^73–75^ It is then possible to identify the CG conformation from the library that is the closest RMSD match with the input conformation and the corresponding rotation and translation transformations that maximize the overlap between the two. The same rotation and translation that maximize the overlap between input and closest reference CG configuration are used to overlap the corresponding atomistic representation to the input. Fig. 3b illustrates the input (hybrid 3 beads and 2 C atoms in orange) overlapped with the output atomistic for seven different cases. The procedure is analogous for the shortest branch of the side chain, where the number of configurations to consider was 243.

The back-mapping procedure was carried out on the last snapshot of an equilibrated hybrid model at 300 K to achieve topologies with all-atom detail. The back-mapped configurations were then used to perform simulations at 300 K for 20 ns of NPT simulation.

## 3 Results

### 3.1 Bonding parameters

**Fig. 4:**
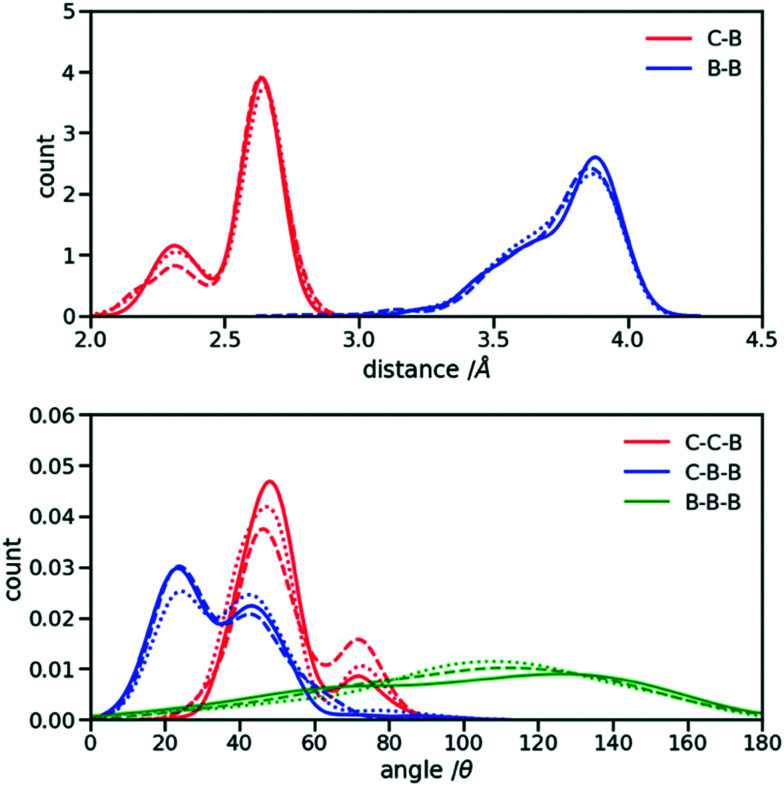
Distribution of the extra bond and angles associated with the CG section with C and B representing carbon atom and bead respectively. 2TT: solid, 4T: dashed and 6T: dotted lines.

The first step for the construction of the hybrid FF is the generation of the bonded parameters. Fig. 4 shows the distribution of all bond distances and angles associated with the coarse-grained beads of the three systems. Different colours are used for different parameters. It is evident that the distributions are nearly identical for different systems. Following eqn (3) the average values and the standard deviation of these distributions were calculated and used to determine the force constant and equilibrium value of the interactions. The FF parameters are consequently very similar, as seen in Table 1, and transferable across different polymers. Therefore, parameters of 2TT were selected to be used across systems for the rest of the simulations.

**Table 1:** Bonded FF parameters for each of the systems

Parameters	2TT	4T	6T
*r*_(0,CB)_ /Å	2.55	2.57	2.55
*σ*_(CB)_ /Å	0.15	0.16	0.16
*K*_(CB)_ /kcal mol^−1^ Å^−2^	25.7	23.7	23.3
*r*_(0,BB)_ /Å	3.76	3.75	3.75
*σ*_(BB)_ /Å	0.19	0.20	0.18
*K*_(BB)_ /kcal mol^−1^ Å^−2^	16.4	14.8	18.3
*θ*_(0,CCB)_ /deg	129	127	130
*σ*_(CCB)_ /deg	10.9	13.0	12.0
*K*_(CCB)_ /kcal mol^−1^ rad^−2^	16.5	11.5	13.4
*θ*_(0,CBB)_ /deg	33.8	34.6	37.1
*σ*_(CBB)_ /deg	14.3	14.1	15.4
*K*_(CBB)_ /kcal mol^−1^ rad^−2^	9.52	9.66	8.25
*θ*_(0,BBB)_ /deg	78.9	77.2	77.6
*σ*_(BBB)_ /deg	39.4	35.0	34.7
*K*_(BBB)_ /kcal mol^−1^ rad^−2^	1.25	1.59	1.62

### 3.2 Non-bonding parameters reproducing the correct density

**Fig. 5:**
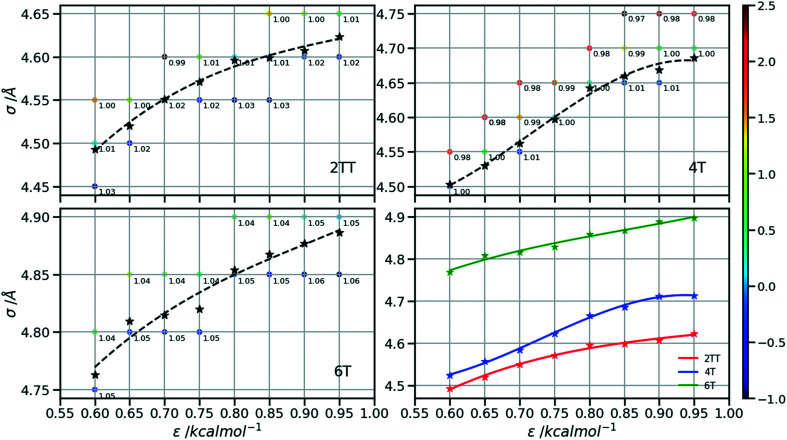
Plots representing the non-bonded pair parameters for which the CG–AA density matches that of the full AA. Points are coloured according to their % difference from the target AA density of each system.

Following the procedure explained in the methods section, the density of the three hybrid AA–CG models was computed for pairs of (*ε*, *σ*) varied over a rectangular grid. Fig. 5 reports the density on each point which is also colour coded according to the distance from the target density computed for the same system in a full AA detail. Interpolation was performed to identify the points where the AA–CG density matched the AA density at certain values of *ε* (indicated with a star in the plot). The interpolating line (dashed black in Fig. 5) connecting these points represents the function *σ*(*ε*) such that the density of the AA–CG coincides with the density of the full AA simulation. Different systems are each shown in individual panels as well as a panel (lower right) that collectively compares the three systems.

The value of *σ* for each *ε* increases going from 2TT to 4T and 6T, *i.e.* with the increase of the length of the rigid conjugated portion and the expected increase of the free volume of the side chain captured by a larger *σ*. The largest difference between 2TT and 6T is around 6% and can be considered too large for a transferrable potential. For this reason, the hybrid simulations in the rest of this work will be carried out considering different parameters for each of the three systems. On the other hand, as we discuss below, any discrepancy in density is removed if one performs a short equilibration run post back-mapping and one may also consider using an average potential across all systems if a back-mapping component is planned in the workflow.

### 3.3 Assessment of hybrid model of monomer simulations

Up to this point of the study, the simple two-parameter hybrid model constrained to reproduce the density of the AA model leaves one degree of freedom to further adjust the model. Since the property of interest (electronic) for these materials has been shown to be directly affected by the conformation of their backbone, we compare the radial distribution function (RDF) of the atomistic parts in both full AA and hybrid models looking for the non-bonded potential parameters that make the two as close as possible. It should be noted that this is uncommon as it is most typical for a CG representation to attempt the best possible description of the atomistic part it replaces, but, as noted in the introduction, the motivation of hybrid models strongly determines the detail of the implementation.^62,69,70^ If, for example, one were interested in another property (*e.g.* compressibility), the non-bonded potential could be set to match that property. For this comparison, three sets of non-bonded pair parameters *ε* = 0.6, 0.75 and 0.95 kcal mol^−1^ and their corresponding *σ* value for each system were considered sampling the same plausible range considered before. Using each set of pair parameters, simulations were performed for 10 ns on systems consisting of 64 monomers (the last 8 ns were used in the analyses). The RDFs are reported between the following atom classes: (i) the carbon atoms in the middle of the DPP unit, labelled as DPP in the figures, (ii) the sulphur atom (S) featuring in all monomers that connect the DPP units as a selection of atoms representing the backbone.

**Fig. 6:**
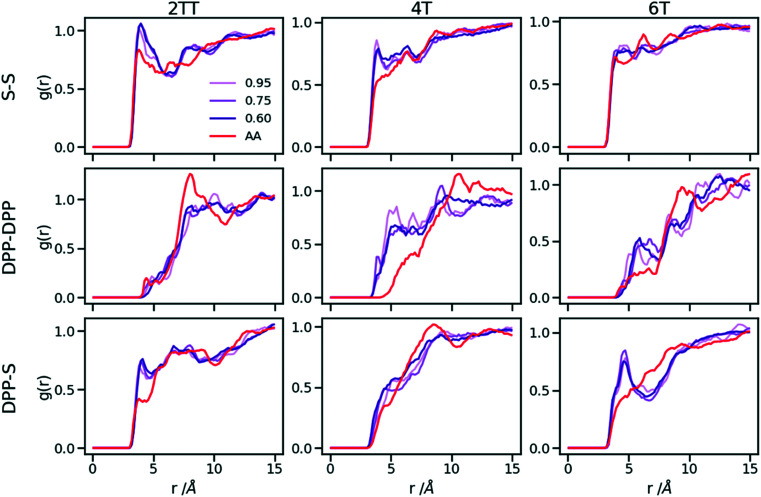
Intermolecular RDFs are plotted for three simulations each using a different set of non-bonded pair values. 0.6, 0.75 and 0.95 kcal mol^−1^ labels correspond to *ε* values.

As evident from Fig. 6, the hybrid model for all the systems shows an increased level of ordering in the backbone atom classes. Across all systems and irrespective of the potential chosen, there are more short-range contacts in the hybrid models with respect to the AA models, which are likely due to the smoother potential of the side chains offering fewer obstacles for the packing of the backbones. The system 4T shows that the DPP–DPP interaction is at a shorter range (before 5 Å) in the hybrid model compared to the AA one explaining the induced ordering. While the decision to use systems of monomers facilitates a quick equilibration, those smaller units can contribute to the introduction of artificial ordering and increased backbone interactions in the absence of the topological constraints imposed by longer chains. This hypothesis was further investigated by comparing the structural properties of larger systems consisting of trimers (in the following section). The hybrid models behave quite similarly to each other but, amongst the three different ε values tested, the higher value of *ε* = 0.95 kcal mol^−1^ results in the highest difference in structural properties to the AA due to the larger bead sizes. As discussed in an earlier article^27^ 4T displays a higher level of conformational flexibility resulting from a comparable volume of side chains and backbone which cause a more homogeneous mixing of side chains and backbones in the atomistic representation. The coarser representation of the side chain facilitates greater segregation of side chains and conjugated backbone, resulting in an increased level of backbone ordering.

The approach used allows the fine-tuning of one parameter of the coarse-grained section. Depending on the desired interaction of the AA to be represented in the hybrid, a pair can be selected that represents that interaction best. The results of Fig. 6 indicate that the discrepancies in RDF originate in large part from the approximated form of the potential and cannot be reduced too much by a suitable choice of parameters. The quality of the hybrid model is similar for the lower two values of *ε* considered and, in the continuation of this work we will study hybrid models with *ε* = 0.75 kcal mol^−1^ and the corresponding *σ* for each system. The hybrid model, evaluated from monomer simulations, seems more promising for 2 of the systems (2TT and 6T). We will consider next the performance of the approach for oligomers and in conjunction with back-mapping.

### 3.4 Assessment of hybrid model of trimer simulations

This work explores the idea of developing coarse-grained potentials generated rapidly from the simulation of monomers for the study of larger oligomers. While the method does not require the availability of equilibrated simulations of oligomers, to evaluate the accuracy of the methodology it is of course useful to compare with a reference equilibrated simulation of oligomers, which, in this case, derives from an earlier study.^27^ However, within the spirit of this approximation, we will not attempt the improvement of the force field *via* re-parameterization of the potential but *via* back-mapping into AA representation.

**Fig. 7:**
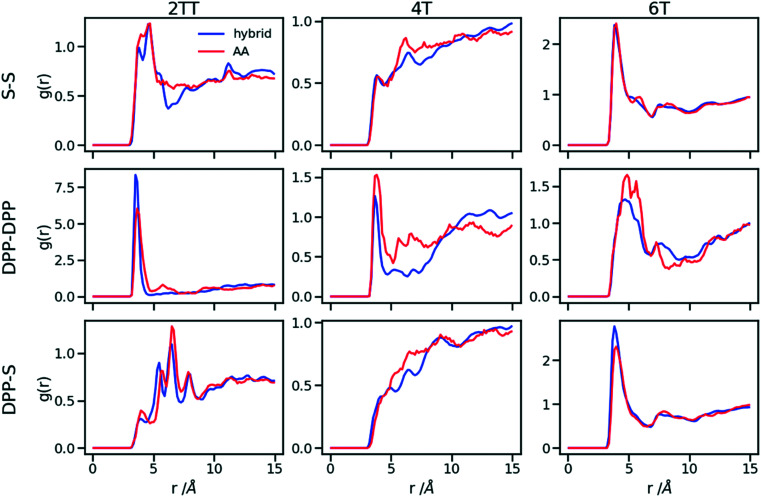
RDFs of the backbone atom classes for the hybrid model *vs.* the AA.

**Fig. 8:**
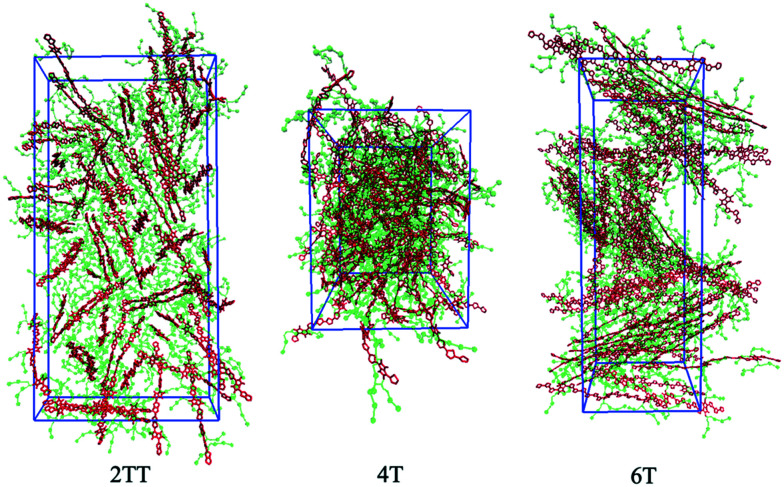
Snapshots of the three hybrid systems where the backbones are depicted in red and the beads in green. Hydrogens are omitted for simplicity.

Hybrid models were pre-equilibrated at 600 K for 30 ns and slowly annealed to 300 K with a cooling rate of 0.01 K ps^−1^. The snapshots used in the analysis section are from equilibration simulations at 300 K for 30 ns. Fig. 7 represents a comparison between the RDFs of the hybrid model and AA for oligomer systems. As shown in Fig. 7, the RDFs of most atom classes of the hybrid model match well with that of the AA using larger systems of oligomers. Somewhat surprisingly, the hybrid model appears to be more accurate for larger systems that were not used in determining its parameters. This is possibly due to the longer chains that prevent the artificial ordering observed in the monomer system. Specifically, the interactions between the rigid backbone of DPP determine local stacking interaction of the π-systems and self-organisation, which reduce the role of the side chains and the effect of the more approximated coarse-grained representation. The largest discrepancy between the two models is observed once again for 4T, but, in this case, the hybrid model display reduced interaction between the backbone atoms with respect to the AA simulation. To illustrate the differences in the level of ordering for different systems, Fig. 8 represents three snapshots of the hybrid models where the ordering increases from 2TT to 6T and 4T.

**Fig. 9:**
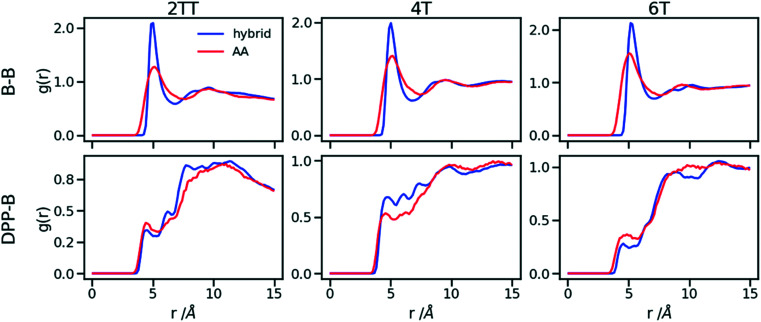
RDFs comparison for the interaction of beads (B) and backbones in hybrid models and as they would be in AA of the system of oligomers.

To understand the origin of the discrepancies between AA and hybrid model for 4T and to offer a more complete description of the hybrid model we report in Fig. 9 the RDF of beads (B) and backbone (represented by carbon atoms in the middle of the DPP unit). The figure compares with AA simulation where there are no beads but coordinates of “virtual” beads can be identified from the atomistic coordinates and have been used to compute the RDF. A first observation is that the B–B potential of the hybrid simulation is narrower than what can be inferred from the RDF of the AA simulation. This aspect could be improved using a separate potential for the bead-bead interaction (like a softer Mie potential^76^ or a numerical potential^31,77–79^) while retaining the computationally convenient Lennard–Jones for the bead-atom interaction. The latter seems indeed fairly accurate for 2TT and 6T. For 4T, this is also acceptable, but we observe a slightly stronger DPP–B interaction in the region between 4 and 8 Å. This is the same region where there is a depletion of the DPP–DPP population in the hybrid with respect to the AA (Fig. 7). We can, therefore, infer that the discrepancy between atomistic and hybrid simulation for 4T is due to an interaction between beads and DPP which is too strong. This cannot be solved within the functional form of the potential explored in this work, but it is possible to consider separate bead–bead and bead-atom potential that would retain most of the advantages of this method with only a moderate increase in effort for parametrizing the potential.

### 3.5 Back-mapping of the hybrid model to all-atom details

**Fig. 10:**
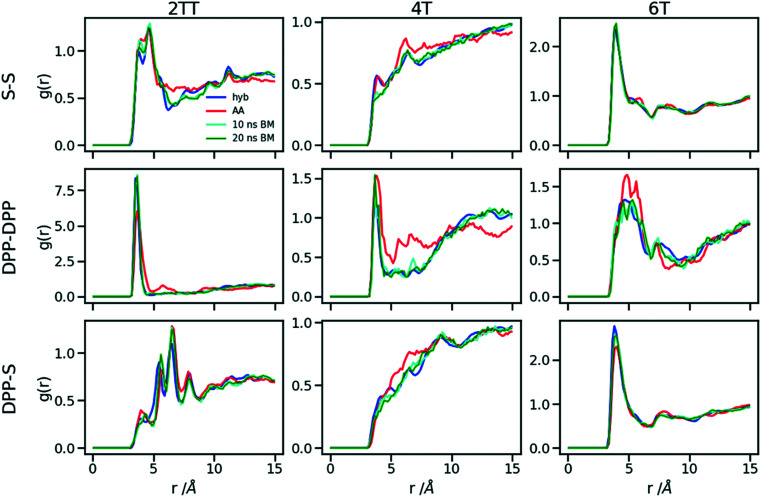
RDFs of the backbone atom classes for the back mapped (BM) systems *vs.* the AA.

A further possible improvement was investigated by performing a back-mapping after the equilibration of the hybrid model to gain all-atom details of the systems. The RDFs of the same atom classes were calculated and plotted in Fig. 10 after back-mapping the hybrid into an atomistic model and equilibrating the system for either 10 or 20 ns (to assess whether a slow equilibration is taking place after back-mapping). The results show an insignificant change in the RDF of the backbone atom classes after back-mapping showing that the hybrid simulations are a good representation of the AA system. Longer equilibrations of the back-mapped model do not change appreciably the RDFs.

Table 2 represents a comparison between the densities of the systems in the two forms of atomistic and hybrid models for oligomer systems. The densities and their standard deviations were calculated from 20 ns of simulation at 300 K after discarding the first 1 ns of simulation. We can see that the density of 6T with the largest chains have the minimum change between full AA and hybrid while being the only system that undergoes a reduction in the density when hybridisation is performed. The other two systems of 2TT and 4T see an increase of density changing from AA to hybrid expected due to the larger sized beads and reduced conformational freedom. We also verified that the average side chain length is similar between the AA and hybrid model (the discrepancy is less than 3%) to rule out radical changes in the mass distribution between the two models. The differences in the density of systems between the hybrid and full AA models are removed after performing the back-mapping and a short equilibration.

**Table 2:** Densities compared for AA and hybrid models of trimer systems as well as after the back-mapping

System	*ρ* AA trimer/g cm^−3^	*ρ* hybrid trimer/g cm^−3^	*ρ* after back-mapping/g cm^−3^
2TT	1.02 ± 0.002	1.08 ± 0.004	1.01 ± 0.002
4T	1.02 ± 0.002	1.06 ± 0.003	1.02 ± 0.002
6T	1.12 ± 0.002	1.11 ± 0.002	1.12 ± 0.002

### 3.6 Electronic structure calculations directly from hybrid model

**Fig. 11:**
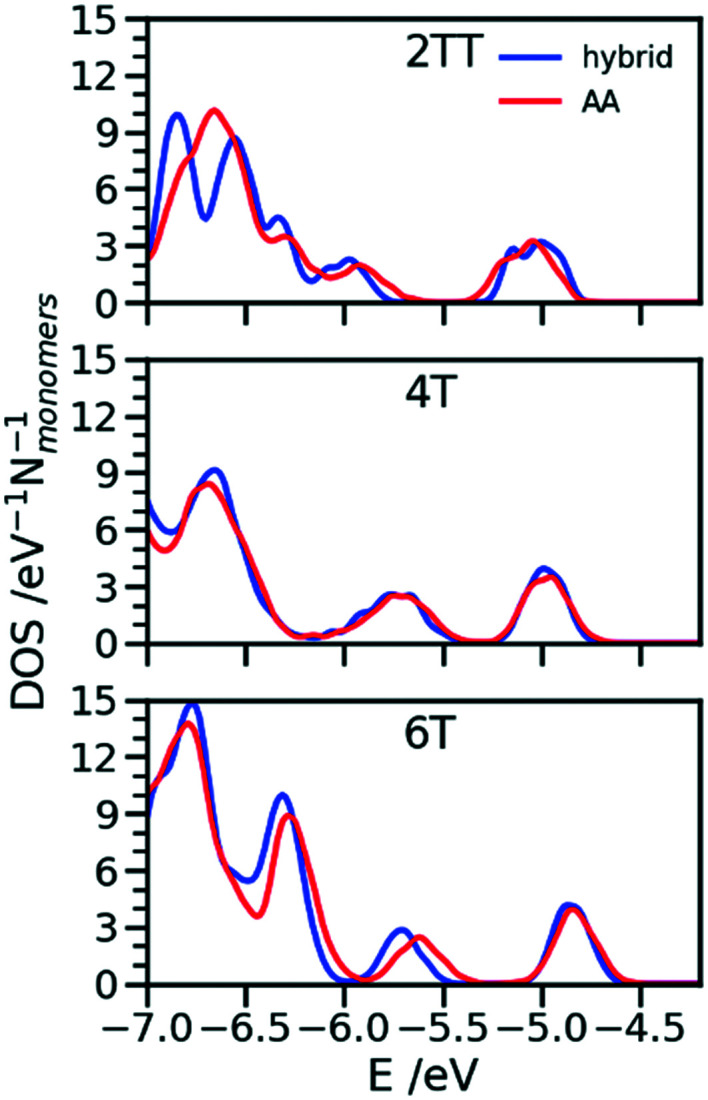
Comparison of DOS computed in the region of the high energy occupier orbital for the three systems using hybrid and AA simulations.

One of the greatest advantages of the hybrid representation is that it retains enough information to enable the calculation of electronic structure since one can safely neglect the contribution of alkyl side chains to the states relevant for charge transport or optical properties. To illustrate this potential application of the hybrid representation we evaluate the electronic density of states (DOS) for the three trimers in the region of the highest occupied orbitals and we compare the results for the hybrid and the AA model. For both of them the calculation was performed for 64 oligomers taken from 4 snapshots of MD separate by 15 ns. The electronic structure was computed on a model where the alkyl side chains have been removed and the dangling bond saturated with a hydrogen atom. The calculations have been performed at the B3LYP/3-21G* level and the DOS reported in Fig. 11 was obtained from the computed orbital levels introducing a broadening of 0.03 eV.

The most important feature of the DOS of the three oligomers is the isolated high energy peak, which corresponds to the highest occupied band. Each trimer contributes to three orbitals in that region, approximately corresponding to a linear combination of the donor fragment DPP.^80^ The DOS computed from atomistic and hybrid model are very similar including the details that are relevant for transport like the small increase in bandwidth in the order 6T < 4T < TT which is due to the increase in super exchange interaction between DPP units with decreasing distance between them. It should be remarked that AA and hybrid simulations have been performed independently, *i.e.* the conformational space explored by the conjugated fragment is similar (for what concerns the orbital properties) whether the overall model is atomistic or hybrid. This is particularly useful as a possible screening of polymers for their electronic properties could be performed using only the hybrid resolution.

## 4 Conclusion

A rapid equilibration technique for simulation of semiconducting polymers was explored in this study using a hybrid model where the alkyl side chains are represented with a coarse-grained potential and the backbone is described with full atomistic details. Three systems with similar side chains have been studied to explore the possible transferability of such potential. The model is constructed to be very easy to parametrize from inexpensive simulations of a solution of monomers. In particular, a two-parameter Lennard–Jones potential describes the non-bonded interactions both between coarse-grained beads and between beads and atoms. By imposing the requirement that hybrid and atomistic models of the monomer have the same density, only one degree of freedom is left in the parametrization of the non-bonded hybrid potential. The best hybrid non-bonded potential is chosen as the one producing a radial distribution function of backbone atoms in best agreement with the atomistic model.

The parameters achieved through this process were further used for the simulation of larger oligomer systems and compared to previously equilibrated full atomistic systems. The agreement between hybrid and atomistic models is very good for two of the three systems and only moderate for one of them, 4T, which is more flexible and displays a greater mixture of backbone and side chain. Interestingly, the quality of the hybrid model improves when longer chains are considered. The reintroduction of atomistic details *via* back-mapping produces an equilibrated structure very similar to those of the hybrid model and an equilibrium density that matches that of the fully atomistic calculations.

For the application of this approach to different materials of this class for which a reference simulation for longer oligomers is not available one can reuse the intramolecular component of the potential, which appears to be transferable. The fine-tuning of the non-bonded potential is inexpensive if one uses simulations of monomers. A very convenient feature is that, from these preliminary simulations alone, it is already possible to assess the expected quality of the hybrid potential when used for larger systems. Finally, the hybrid representation retains the information required to evaluate the electronic structure of the conjugated backbone and can be used in conjunction with electronic structure methods.

## Conflicts of interest

There are no conflicts to declare.

## Supplementary Material
